# Supplementary material for: Gut microbiota of endangered Australian sea lion pups is unchanged by topical ivermectin treatment for endemic hookworm infection
Source: Front Microbiol. 2022 Dec 19;13:1048013. doi: 10.3389/fmicb.2022.1048013 (PMC9806137; doi:10.3389/fmicb.2022.1048013)
Supplement: Supplementary file 1 [file Data_Sheet_1.PDF]

Supplementary Table 1. Results from linear mixed models one and two, analysing the correlations between pup morphometric and haematological data and alpha diversity in the 2019 and 2020/21 breeding seasons.

Alpha diversity metric	Model	Term	Est	S.E.	Lower CI	Upper CI	p-value	R^2 Value	Breeding Season
Chao1	1	Treatment group	8.65	10.12	-11.61	28.78	0.395		
		Capture	-23.9	12.28	-48.24	0.51	0.048*		
		Age	17.01	9.50	-3.49	35.83	0.076	0.086	2019
		Weight	4.46	3.48	-2.43	11.36	0.203		
		Standard length	-1.99	1.96	-5.87	1.89	0.312		
		Sex	-4.58	10.73	-25.9	16.80	0.670		
Chao1	2	Treatment group	18.785	11.848	-4.685	42.255	0.14		
		Capture	-35.451	13.094	-61.390	-9.513	0.013*		
		Age	20.959	9.358	2.422	39.496	0.08		
		Hookworm status	10.621	19.949	-28.894	50.138	0.5		
		Total plasma protein	1.745	0.894	-0.026	3.517	0.09	0.134	2019
		Total nucleated cell count	-1.860	7.959	-17.627	13.906	0.82		
		Absolute neutrophil count	0.595	8.262	-15.770	16.969	0.95		
		Absolute lymphocyte count	3.841	9.265	-14.505	22.189	0.69		
		Absolute monocyte count	5.565	17.119	-28.344	39.475	0.76		
		Absolute eosinophil count	-6.883	12.341	-31.330	17.563	0.61		
Shannon-Wiener Index	1	Treatment group	-2.430	0.153	-0.553	0.069	0.124		
		Capture	0.119	0.173	-0.234	0.455	0.527		
		Age	-0.127	0.137	-0.410	0.145	0.357	0.097	2019
		Weight	0.101	0.049	-0.004	0.199	0.072		
		Standard length	-0.049	0.027	-0.104	0.005	0.076		
		Sex	-0.279	0.162	-0.607	0.051	0.096		
Shannon-Wiener Index	2	Treatment group	-0.154	0.201	-0.530	0.218	0.436		
		Capture	-0.199	0.204	-0.633	0.127	0.225		
		Age	0.041	0.149	-0.235	0.320	0.783		
		Hookworm status	-0.247	0.301	-0.812	0.310	0.414		
		Total plasma protein	-0.021	0.013	-0.005	0.045	0.148	0.089	2019
		Total nucleated cell count	-0.093	0.118	-0.312	0.130	0.430		
		Absolute neutrophil count	0.077	0.122	-0.152	0.304	0.527		
		Absolute lymphocyte count	0.125	0.139	-0.134	0.386	0.372		
		Absolute monocyte count	0.112	0.255	-0.394	0.586	0.662		
		Absolute eosinophil count	0.018	0.184	-0.335	0.358	0.919		
Chao1	1	Treatment group	13.320	7.086	-0.723	27.360	0.043*	0.107	2020
		Capture	6.494	9.333	-12.00	24.989	0.488		
		Age	1.366	8.018	-26.673	5.106	0.182		

		Weight	0.863	2.490	-4.072	5.799	0.729		
		Standard length	0.840	1.511	-2.155	8.384	0.579		
		Sex	1.366	9.230	-16.924	19.657	0.882		
Chao1	2	Treatment group	19.575	7.918	3.883	35.267	0.015*		
		Capture	16.512	8.061	0.548	32.485	0.435		
		Age	-10.890	7.008	-24.777	2.996	0.123		
		Hookworm status	15.412	10.484	-5.361	36.187	0.145		
		Total plasma protein	0.833	0.713	-0.579	2.246	0.245	0.135	2020
		Total nucleated cell count	-1.813	2.515	-6.799	3.171	0.472		
		Absolute neutrophil count	1.565	2.638	-3.661	6.793	0.554		
		Absolute lymphocyte count	2.536	5.076	-7.522	12.596	0.618		
		Absolute monocyte count	0.278	12.016	-23.532	24.089	0.981		
		Absolute eosinophil count	3.259	7.232	-11.071	17.590	0.653		
Shannon-Wiener Index	1	Treatment group	0.573	0.151	0.272	0.876	0.002*		
		Capture	-0.428	0.200	-0.826	-0.031	0.350		
		Age	0.035	0.171	-0.307	-0.374	0.846	0.198	2020
		Weight	0.027	0.053	-0.078	0.133	0.610		
		Standard length	0.033	0.032	-0.305	0.097	0.301		
		Sex	0.158	0.197	-0.233	0.551	0.425		
Shannon-Wiener Index	2	Treatment group	0.590	0.172	0.249	0.935	0.04*		
		Capture	-0.204	0.175	-0.553	0.142	0.28		
		Age	0.198	0.152	-0.103	0.508	0.23		
		Hookworm status	0.180	0.228	-0.271	0.632	0.45		
		Total plasma protein	-0.009	0.015	-0.040	0.021	0.55	0.200	2020
		Total nucleated cell count	-0.066	0.054	-0.175	0.041	0.26		
		Absolute neutrophil count	0.083	0.057	-0.029	0.197	0.17		
		Absolute lymphocyte count	0.115	0.110	-0.103	0.334	0.34		
		Absolute monocyte count	0.210	0.261	-0.313	0.728	0.47		
		Absolute eosinophil count	0.007	0.157	-0.304	0.319	0.98		

Supplementary Table 2. Results from linear mixed models three and four, analysing the correlations between pup morphometric and haematological data and alpha diversity within each treatment group in 2019 and 2020/21.

Alpha diversity metric	Model	Term	Est	S.E.	Lower CI	Upper CI	p-value	R^2 Value	Treatment group	Breeding Season
Chao1	3	Capture	0.030	0.202	-0.377	0.441	0.879			
		Age	0.050	0.180	-0.411	0.413	0.782			
		Weight	0.060	0.076	-0.093	0.234	0.435	0.038	Untreated (control)	2019
		Standard length	-0.036	0.037	-0.112	0.039	0.344			
		Sex	-0.143	0.207	-0.583	0.323	0.507			
Chao1	4	Capture	-0.226	0.225	-0.676	0.229	0.319			
		Age	0.045	0.154	-0.274	0.353	0.768			
		Hookworm status	0.204	0.285	-0.367	0.776	0.477			
		Total plasma protein	0.022	0.014	-0.005	0.051	0.118			
		Total nucleated cell count	-0.360	0.281	-0.925	0.202	0.206	0.268	Untreated (control)	2019
		Absolute neutrophil count	0.303	0.287	-0.270	0.877	0.295			
		Absolute lymphocyte count	0.306	0.293	-0.28	0.893	0.301			
		Absolute monocyte count	0.007	0.360	-0.712	0.741	0.983			
		Absolute eosinophil count	0.248	0.322	-0.395	0.902	0.443			
Shannon-Wiener Index	3	Capture	-39.300	14.929	-69.169	-9.432	0.011*			2019
		Age	31.339	13.346	4.634	58.039	0.231			
		Weight	8.328	5.675	-3.025	19.681	0.148	0.176	Untreated (control)l	
		Standard length	-4.191	2.797	-9.786	1.405	0.140			
		Sex	20.705	15.281	-9.871	51.276	0.181			
Shannon-Wiener Index	4	Capture	-43.716	18.096	-79.920	-7.510	0.019*			
		Age	21.668	12.389	-3.118	46.453	0.086			
		Hookworm status	13.920	22.988	-32.071	59.911	0.544			
		Total plasma protein	2.620	1.155	0.308	4.931	0.028			
		Total nucleated cell count	9.467	22.661	-35.890	54.804	0.678	0.257	Untreated (control)	2019
		Absolute neutrophil count	-12.294	23.069	-58.448	33.86	0.596			
		Absolute lymphocyte count	-14.615	23.604	-61.838	32.609	0.538			
		Absolute monocyte count	-13.617	28.956	-71.549	44.314	0.640			
		Absolute eosinophil count	-14.801	25.897	-66.614	37.011	0.570			
Chao1	3	Capture	-7.996	22.199	-52.655	37.272	0.720			
		Age	4.020	13.585	-25.681	31.609	0.769			
		Weight	3.184	4.363	-5.594	12.023	0.471	0.105	Treated	2019
		Standard length	-1.256	0.293	-7.505	4.868	0.671			
		Sex	-26.477	15.211	-58.206	7.457	0.110			
Chao1	4	Capture	-14.720	18.031	-52.840	23.586	0.419			
		Age	11.611	13.451	-16.746	39.684	0.393			

		Hookworm status	-22.551	35.06	-93.422	47.645	0.523			
		Total plasma protein	-0.922	1.723	-4.669	2.773	0.595			
		Total nucleated cell count	-6.196	7.816	-22.181	10.651	0.433	0.158	Treated	2019
		Absolute neutrophil count	5.723	8.369	-12.581	22.917	0.498			
		Absolute lymphocyte count	14.236	10.378	-6.798	25.104	0.177			
		Absolute monocyte count	28.268	23.496	-20.780	76.071	0.236			
		Absolute eosinophil count	-9.502	16.635	-43.031	23.966	0.571			
Shannon-Wiener Index	3	Capture	0.209	0.324	-0.450	0.874	0.526			
		Age	-0.303	0.198	-0.700	0.099	0.135			
		Weight	0.153	0.063	0.021	0.280	0.075	0.203	Treated	2019
		Standard length	-0.073	0.043	-0.162	0.014	0.097			
		Sex	-0.418	0.217	-0.871	0.048	0.075			
Shannon-Wiener Index	4	Capture	-0.191	0.264	-0.722	0.343	0.473			
		Age	0.036	0.197	-0.360	0.341	0.854			
		Hookworm status	-0.998	0.524	-2.166	0.086	0.064			
		Total plasma protein	-0.003	0.024	-0.054	0.046	0.883			
		Total nucleated cell count	-0.056	0.116	-0290	0.180	0.629	0.089	Treated	2019
		Absolute neutrophil count	0.073	0.125	-0.178	0.324	0.558			
		Absolute lymphocyte count	0.159	0.153	-0.148	0.468	0.303			
		Absolute monocyte count	0.413	0.349	-0.334	1.138	0.242			
		Absolute eosinophil count	0.072	0.249	-0.430	0.597	0.771			
Chao1	3	Capture	12.171	12.632	-13.261	37.494	0.340			
		Age	-26.853	11.555	-50.016	-3.665	0.249			
		Weight	4.099	3.135	-2.187	1.384	0198	0.260	Untreated (control)	2020
		Standard length	0707	1.894	-3.089	4.53	0.710			
		Sex	3.958	12.470	-21.028	-3.665	0.752			
Chao1	4	Capture	37.173	10.998	17.887	56.470	0.186			
		Age	-33.676	10.308	-51.778	-15.570	0.254			
		Hookworm status	32.174	12.566	10.107	-54.242	0.152			
		Total plasma protein	1.551	0.918	-0.060	3.163	0.100			
		Total nucleated cell count	-7.047	4.985	-15.802	1.707	0.166	0.341	Untreated (control)	2020
		Absolute neutrophil count	8.247	4.245	0.791	15.703	0.060			
		Absolute lymphocyte count	5.7773	8.495	-9.141	20.696	0.501			
		Absolute monocyte count	-18.983	24.659	-62.269	24.302	0.466			
		Absolute eosinophil count	-1.135	11.937	-22.098	19.826	0.924			
Shannon-Wiener Index	3	Capture	-0.668	0.343	-1.370	0.019	0.0681			
		Age	-0.079	0.314	-0.709	9.557	0.802			
		Weight	0.063	0.085	-0.107	0.234	0.457	0.177	Untreated (control)	2020
		Standard length	0.518	0.051	-0.051	0.155	0.319			

		Sex	0.162	0.314	-0.519	0.842	0802			
Shannon-Wiener Index	4	Capture	-0.116	0.288	-0.775	0.462	0.690	0.208	Untreated (control)	2020
		Age	0.105	0.270	-0.440	0.764	0.701			
		Hookworm status	0.505	0.326	-0.157	1.185	0.129			
		Total plasma protein	-0.028	0.023	-0.079	.0205	0.247			
		Total nucleated cell count	-0.149	0.129	-0.411	0.120	0.256			
		Absolute neutrophil count	0.216	0.110	-0.010	0.439	0.056			
		Absolute lymphocyte count	0.095	0.220	-0.361	0.543	0.668			
		Absolute monocyte count	0.347	0.640	-0.938	1.632	0.590			
		Absolute eosinophil count	0.104	0.310	-0.545	0.734	0.737			
Chao1	3	Capture	1.536	13.313	-25.098	28.171	0.115	0.045	Treated	2020
		Age	1.082	10.480	-19.885	22.049	0.103			
		Weight	-3.236	3.553	-10.345	3.872	-0.911			
		Standard length	2.134	2.264	-2.394	6.663	0.943			
		Sex	4.127	10.480	-20.874	29.126	0.103			
Chao1	4	Capture	2.748	12.8-8	-22.877	28.375	0.831	0.084	Treated	2020
		Age	1.272	11.554	-21.843	24.388	0.913			
		Hookworm status	-23.428	21.450	-66.344	19.488	0.280			
		Total plasma protein	-0.363	1.454	-3.274	2.546	0.804			
		Total nucleated cell count	-2.375	3.657	-9.693	2.942	0.519			
		Absolute neutrophil count	2.822	4.206	-5.594	11.238	0.506			
		Absolute lymphocyte count	8.471	7.407	-6.348	23.292	0.259			
		Absolute monocyte count	-0.859	15.121	-31.113	29.394	0.955			
		Absolute eosinophil count	10.682	9.412	-8.149	29.513	0.262			
Shannon-Wiener Index	3	Capture	-0.209	0.218	-0.640	0.234	0.358	0.029	Treated	2020
		Age	0.173	0.172	-0.170	0.517	0.319			
		Weight	-0.020	0.058	-0.137	0.096	0.725			
		Standard length	0.017	0.037	-0.057	0.091	0.643			
		Sex	0.035	0.205	-0.374	0.448	0.863			
Shannon-Wiener Index	4	Capture	-0.247	0.203	-0.655	0.160	0.231	0.127	Treated	2020
		Age	0.216	0.183	-0.151	0.593	0.244			
		Hookworm status	-0.161	0.341	-0.833	0.520	0.637			
		Total plasma protein	0.023	0.023	-0.022	0.070	0.307			
		Total nucleated cell count	-0.036	0.058	-0.152	0.080	0.534			
		Absolute neutrophil count	0.038	0.066	-0.095	0.172	0.568			
		Absolute lymphocyte count	0.119	0.117	-0.116	0.355	0.315			
		Absolute monocyte count	0.140	0.240	-0.348	0.621	0.561			
		Absolute eosinophil count	0.066	0.149	-0.233	0.365	0.661			
